# Multi-Unmanned Aerial Vehicle (UAV) Cooperative Fault Detection Employing Differential Global Positioning (DGPS), Inertial and Vision Sensors

**DOI:** 10.3390/s90907566

**Published:** 2009-09-24

**Authors:** Guillermo Heredia, Fernando Caballero, Iván Maza, Luis Merino, Antidio Viguria, Aníbal Ollero

**Affiliations:** 1 Robotics, Vision and Control Group, University of Seville, Camino de los Descubrimientos s/n, 41092, Seville, Spain; webpage: http://grvc.us.es; 2 Pablo de Olavide University, Crta. Utrera km. 1, 41013 Seville, Spain; 3 Centro Avanzado de Tecnologías Aeroespaciales (CATEC), Aeropolis, 41309 Seville, Spain; E-Mail: aollero@cartuja.us.es (A.O.)

**Keywords:** UAVs, fault detection and identification, multi-UAV, vision sensors, GPS

## Abstract

This paper presents a method to increase the reliability of Unmanned Aerial Vehicle (UAV) sensor Fault Detection and Identification (FDI) in a multi-UAV context. Differential Global Positioning System (DGPS) and inertial sensors are used for sensor FDI in each UAV. The method uses additional position estimations that augment individual UAV FDI system. These additional estimations are obtained using images from the same planar scene taken from two different UAVs. Since accuracy and noise level of the estimation depends on several factors, dynamic replanning of the multi-UAV team can be used to obtain a better estimation in case of faults caused by slow growing errors of absolute position estimation that cannot be detected by using local FDI in the UAVs. Experimental results with data from two real UAVs are also presented.

## 1. Introduction

Unmanned aerial vehicles (UAVs) and aerial robotics have attracted significant interest for a wide range of applications [1]. In many of them, the active cooperation of several UAVs may have important advantages [2]. Reliability is a key issue in aerial vehicles, where Fault Detection and Identification (FDI) techniques play an important role in the efforts to increase the reliability of the system. Most FDI applications to UAVs that appear in the literature use model-based methods, which try to diagnose faults using the redundancy of some mathematical description of the system dynamics. FDI has been applied to unmanned aircraft, either fixed wing UAVs [3] or helicopter UAVs [4–6].

Differential GPS (DGPS) receivers are able to achieve accuracies of a few centimeters using carrier-phase measurements, which make them a good choice for UAV positioning main sensors. Since they usually are the only absolute position sensors employed in UAVs, the reliability of their measurements is critical for UAV missions. If DGPS readings are erroneous or differential mode is lost, position estimation may accumulate large drift errors.

The literature concerning GPS fault detection is extensive; there exist several techniques developed to detect faulty measurements in the receiver, known as Receiver Autonomous Integrity Monitoring (RAIM) [7,8]. However, RAIM techniques need additional satellites in view to operate, and they are difficult to find in commercial GPS receivers. RAIM techniques are usually implemented in aeronautical-grade GPS receivers, which in general can not be used in small and medium UAVs due to their size, weight and cost limitations. This paper will concentrate in FDI for DGPS when RAIM techniques cannot be used.

Ideally, FDI uses all available information to detect malfunctions in UAV subsystems. But there exist positioning errors that cannot be detected using the navigation sensors onboard the UAV. In multi-UAV missions, it is possible to take advantage of the capabilities that the team of UAVs offers to augment each of the individual FDI systems. In this way, sensors from other UAVs can be used to obtain additional data which can be applied to the UAV FDI system to detect faults in its own sensors.

If the UAVs are equipped with visual cameras, different UAVs may identify, using their cameras, common objects in the scene. For instance, the use of a robust feature extraction technique capable of identifying natural landmarks of the scene [2], and the correspondences between the same landmarks obtained by two UAVs provide the relative pose displacement between both UAVs. Thus, for example, if the DGPS of UAV-A is faulty, this fault can be detected by using the DGPS of UAV-B and the relative position estimation computed from the images. The proposed idea is to estimate the position of UAV-A using the known position of UAV-B and the estimation of the relative position of UAV-A and UAV-B using the method described above. Unfortunately, these vision-based position estimations have different accuracy and noise levels depending on several factors. Therefore, a variable threshold strategy, for the fault detection process, has been adopted in this paper. Furthermore, in multi-UAV missions, the probability of having the same scene in the field of view of two or more UAVs when executing a plan is not very high. Thus, in this paper we propose the application of replanning techniques with the automatic generation of new tasks for the multi-UAV team in such a way that the above required condition for fault detection is satisfied for two UAVs. Particularly, the application of a market-based approach for the *multi-robot task allocation* (MRTA) problem is proposed.

The paper is organized as follows. Section 2 presents the techniques for fault detection and identification in helicopter UAVs. Section 3 describes multi-UAV vision-based relative position estimation. Section 4 presents the method implemented to dynamically replan the mission in order to improve the accuracy of the estimation. Section 5 presents the experimental data and the obtained results. Finally, the conclusions and future trends mentioned in Section 6 complete the paper.

## 2. Sensor FDI in Small Autonomous Helicopters

Several small autonomous helicopter prototypes have been developed in recent years at different research centers throughout the world [1]. Autonomous helicopter flight requires precise position and attitude information for control and stabilization. Small autonomous helicopters carry a pack of sensors that in a typical case includes an inertial measurement unit (IMU) with three gyros, three accelerometers and a 3-axis magnetometer for attitude determination, a centimeter-precision kinematic DGPS and an ultrasonic or barometric altitude sensor for take-off and landing. A fault in one of the sensors, if undetected, may induce position and attitude estimation errors. Reconfiguration in these cases usually consists in isolating the faulty sensor and using the other sensors to get the best estimation of position and attitude.

### 2.1. Inertial, magnetometer and altitude sensor faults

Usual UAV sensor faults are additive (sensor output has a drift term added), multiplicative (sensor output is scaled by a multiplicative term) or “stuck” output (sensor output remains fixed at a constant value). The presence of these faults can be detected in most cases by means of the so-called residuals, i.e., quantities that are over-sensitive to the malfunctions. Observer-based and parameter estimation are the most frequently applied methods for residual generation in FDI [9]. Most published work in recent years on FDI systems for autonomous vehicles also use observer-based methods. Neural networks have also been used to detect sensor and actuator faults in aircrafts [10] and UAVs [4,11].

**Figure 1.**
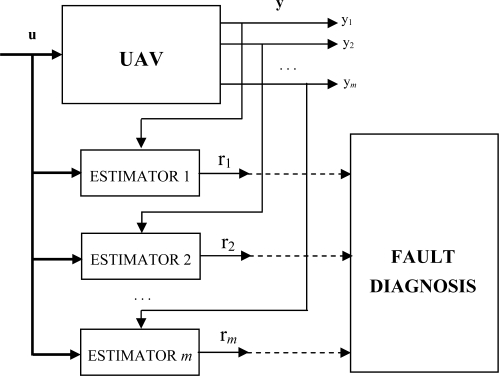
Bank of estimators for output residual generation.

The FDI system implemented in individual helicopters is described in detail in [5]. The structure of the sensor FDI system is based on a bank of output estimators as shown in Figure 1. The number of these estimators is equal to the number of system outputs. A residual is generated for each sensor, comparing the estimator output with the sensor output. Each residual is not affected by the other sensors, and therefore fault identification is straightforward: each residual is only sensitive to a single helicopter sensor.

The FDI system with the above structure has been implemented using ARX input-output estimators. A number of ARX Multi-Input Single-Output (MISO) models have been identified from input-output data. These models are of the type:

(1) $$y_{i}^{*}(t)=\sum_{j=1}^{n}\alpha_{i,j}\;y_{i}^{*}(t-j)+\sum_{j=1}^{r}\sum_{k=1}^{n}\beta_{i,j,k}u_{j}^{*}(t-k)+\varepsilon_{i}(t)$$

The number of identified MISO ARX models is equal to the number *m* of the output variables. The model order *n* and the parameters *α_i,j_* anḍ *β_i,j,k_* with *i* = 1, …, *m*, of the model have to be determined by the identification approach. The term *ε_i_*(*t*) takes into account the modeling error, which is due to process noises, parameter variations, etc. The ARX models are chosen with the structure that achieve the smallest Akaike's Information Theoretic Criterion (AIC) [12], according to a simple search algorithm, in which the first half of data is used for estimation and the second for cross validation.

Independent residuals are constructed for each different sensor failure. Residuals are designed so that they respond to an individual failure and not to the others. In general, residuals *r_k_* are functions of the squared difference between real (*c_i_*) and estimated (*ĉ_i_*) sensor outputs:

(2) $$r_{k}=\sum_{i=1}^{n}m_{i}{\left(c_{i}-{\hat{c}}_{i}\right)}^{2}$$

where *m_i_* are weighting coefficients that are determined for each failure based on experience and experimentation. Ideally, if no fault is present, the residual would be zero. In practice, the residual will take non-zero values due to estimation errors, sensor noise, etc. Usually, the residual for a specific sensor will be bounded, and therefore a “threshold level” can be defined so that the residual is always below it in absence of failures. The system has been tested with different sensors and failure types. The implemented sensor FDI system is able to detect many of these errors.

### 2.2. Differential GPS sensor failures

Consider now the Differential GPS absolute position sensor. This sensor implements complex processing algorithms to generate absolute position estimations from the received satellite signals and the differential corrections. There are several typical GPS error sources such as atmospheric delays, ephemeris errors, satellite and receiver clock errors or multipath signal reception. Many of these errors can be avoided in differential operation.

There are also “system errors”, errors in the space and control segment (satellite hardware failures, control segment software or transmission errors, etc). Although these errors are rare, they can lead to large position errors. These errors have received much attention since the selective availability turning off in 2000, due to the increasing use of GPS in critical civilian applications such as airport approach or coast navigation. These errors can be partially avoided in differential operation.

From a practical point of view, a commercial GPS receiver operating in differential mode may have different error sources, which can be classified in three groups. The first group includes errors that can be detected by the GPS receiver. Among these are satellite signal reception problems, due to intermittent signal blocking when the vehicle is moving because of buildings, foliage and hilly terrain. Another source is differential correction errors. Timely differential corrections are very important for DGPS, but in some cases the correction messages may arrive “corrupted” due to problems on the communication link. Most DGPS receivers can provide an estimation of the level of accuracy of the calculated position, although this accuracy estimation can be erroneous in some cases.

The second group includes errors that cannot be detected by the receiver, but can be detected using vehicle dynamics and model-based fault detection techniques. When in operation, position estimations from DGPS receivers may present *a priori* unknown-source errors. For example, typical results obtained in 24-hour static tests show that estimated position error was less than 2 cm most of the time, but also include several groups of 2 to 5 contiguous points with a 20–60 cm error, which appear from time to time with no predictable frequency, which seems to be caused by RTK integer ambiguity [13], and a few isolated points with large position errors. The number of satellites in view, CRC redundancy code, and indicators of differential carrier-phase estimation were all correct. A possible explanation can be receiver algorithm errors. In [14] another error source is suggested: GPS receivers have thousands of lines of code, many of which are from legacy code that is not fully tested. This can lead to unpredictable errors. Some of the described errors can be detected using model-based fault detection and identification techniques, as described in Section 2.2. Using this information, the faulty GPS estimations can be discarded.

**Figure 2.**
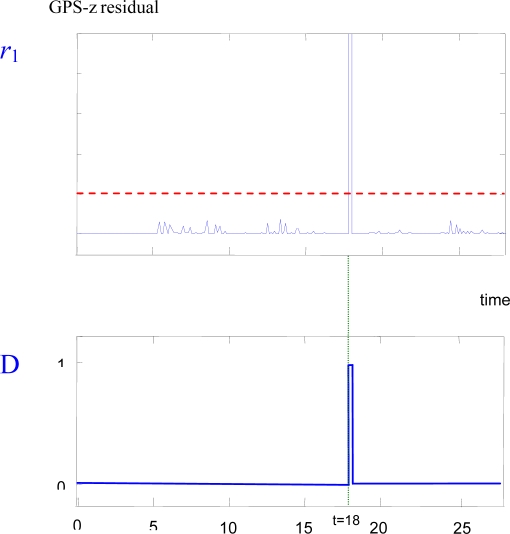
Outlier GPS-z sensor failure detection. Upper plot: residual signal. Lower plot: fault detection signal.

Figure 2 shows the detection of a one-meter outlier-type fault in the GPS-z sensor of the helicopter. In the upper plot of Figure 2 the residual signal of the GPS-z sensor (*r*_1_) generated by the ARX estimator is shown. The fault has been declared at *t* = 18 sec. Shortly after the fault, the residual goes above the threshold value (dashed line). The lower plot shows the fault detection signal (*D*, which equals 1 when a fault is detected, and is zero otherwise).

**Figure 3.**
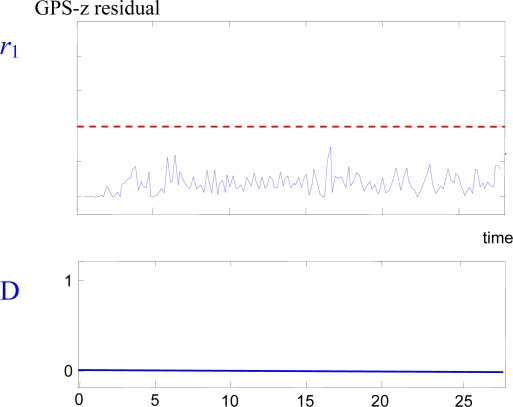
Slow-growing drift GPS-z sensor failure detection. Upper plot: residual signal *r*_1_. Lower plot: fault detection signal.

But there are some cases in which the individual helicopter FDI system cannot detect the failure. For example, slow growing errors in absolute positioning estimations (due to loss or corruption of differential corrections unnoticed by the receiver) are difficult to detect using incremental sensors as gyros or accelerometers, since DGPS is the only absolute positioning sensor that is used in UAVs. Figure 3 shows an example of this case: at *t* = 2 s, a slow growing error is present in the GPS-z sensor, but the FDI system is not able to detect it using the other onboard sensors.

## 3. Multi-UAV Vision-Based Relative Position Estimation

A method for the estimation of the ego-motion of a single UAV by means of monocular vision has been presented in [15]. This method assumes that the imaged scene is approximately flat or, in full 3D environments, that the UAV flies at relative high altitude compared to the deviations from the flat model. Its robustness would be reduced if the UAVs are flying at low altitudes in urban areas or hilly terrains, so the planning system should take this issue into account.

The estimated position may present drifts along the sequence of images. This effect is mainly derived from the accumulative errors in the homography computation used to estimate the planar motion. Mosaicking can help to reduce the registration errors and, hence, to increase the accuracy of the position estimation.

This method can be extended to estimate the relative position among several UAVs. Thus, if two UAVs are registering approximately the same scene and it is possible to match a set of features between their images, the relative displacement between the UAVs can be obtained by computing the plane-induced homography matrix that relates their cameras.

**Figure 4.**
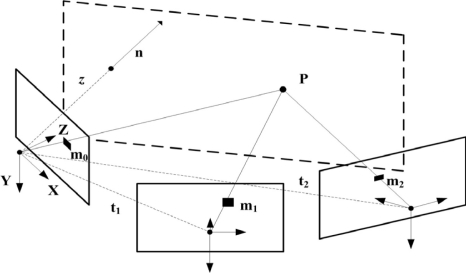
Scheme of the geometry of multiple views of the same plane z.

The technique described in [15] obtains the relative displacement between two views of the same planar scene. The relations presented also hold if the views are taken by different calibrated cameras. In this case, the homography that relates both views, 1 and 2, of the same planar scene (see Figure 4) is given by:

(3) $$H^{12}=A_{1}\cdot R_{2}\cdot \left(I-wt_{2}n^{T}\right)\cdot A_{2}{}^{-1}$$

where **A**_1_ and **A**_2_ are the intrinsic calibration matrices of both cameras, **t**_2_ is the relative translation of the second camera point of view in the first camera coordinate frame, **n** is an unitary vector normal to the plane in the first camera coordinate frame (in the outward camera direction), *w* = 1/*z*, *z* being the distance from the first camera to the plane and **R**_2_ is the rotation matrix that transforms a vector in the first camera coordinate frame into a vector expressed in the second camera coordinate frame. Thus, the relative displacement between two UAVs is obtained by computing the plane-induced homography matrix between their cameras (**H**^12^).

The computation of matches between images taken from very different points of view may be a hard limitation of this approach. Robust and repeatable features, with some degree of invariance, should be used, as affine invariant neighborhoods or maximally stable extreme regions. In our implementation, the algorithm proposed in [2] for blob matching and robust homography estimation is used. The idea is to use this algorithm to compute the homography that relates two images of the same scene taken by different UAVs.

Thus, it is assumed that at a certain time instant (the time of image 0) the same scene is viewed by two UAVs, for instance UAV 1, which is taken as the reference UAV, and UAV 2. The robust matching procedure is applied with the aim to match image 0 of the reference UAV with its most recent captured image of UAV 2. If a number of good blob matches are obtained, the relative displacement between UAV 1 and UAV2 at that time is computed by means of the motion estimation algorithm. Otherwise, the images are discarded.

**Figure 5.**
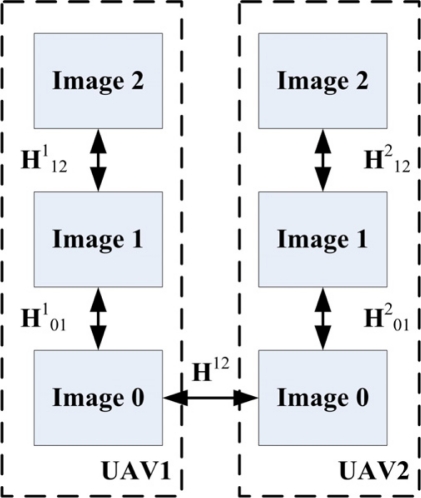
Scheme of relations between homographies.

In general, the probability of having the same scene in the field of view of two or more UAVs is not very high. In Figure 5 **H**^12^ is the homography matrix that relates two images gathered by UAVs 1 and 2 for image 0 computed by blob matching. **H***^j^*_0_*_i_*, the homography matrix between image *i* and image 0 for UAV *j* is computed by composing the homography matrices between consecutive images. For instance, **H***^2^*_02_ = **H***^2^*_01_ **H***^2^*_1_*_2_*. Combining the homographies **H***^2^*_0_*_i_* with **H**^12^ determines the homography matrices of UAV 2 at different time instants along its trajectory with respect to the view of UAV 1 at image 0.

If the position of the reference UAV in a global frame is known, it will be easy to estimate the position of all the UAVs in the global frame. For instance, the location of UAV 2 at time of image 2 with respect the reference UAV at time of image 0 is given by **H**^12^**H**^2^_01_**H**^2^_12_. More details are given in [2].

Assume that a UAV team is executing a mission, for example a surveillance mission. One of the purposes of this work is to outline the replanning necessary to achieve that one UAV estimates the relative position of other UAV applying the method described above. As stated before, it is necessary that both UAVs take images of approximately the same scene.

**Figure 6.**
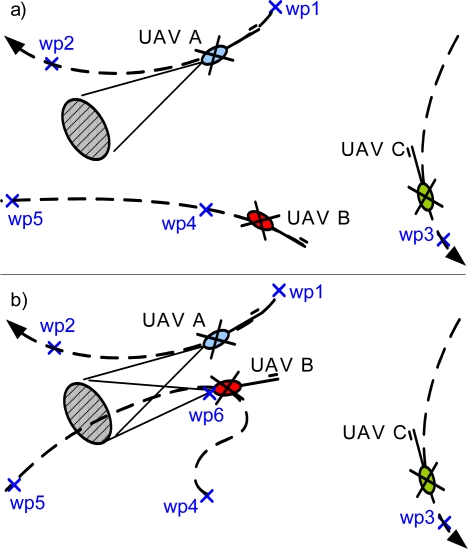
An example illustrating the re-planning process: (a) UAVs following the initial plan. (b) Dynamic replanning: UAV B plan changes to include wp6.

Given some invariant characteristic of the features used to establish matches between the two images [16], it is possible to face significant rotations, translations and scale shifts. However, in order to reduce the complexity of the matching stage, it is recommended to locate the two UAVs at approximately the same altitude (to reduce the scale shift effect) performing an angle of no more that 45 degrees (see Figure 6.b).

In addition, the uncertainty of the computed homography can be used as a measure of the accuracy of the relative position estimation. Particularly, the standard deviation of two parameters related with the uncertainty in the computed translation in pixel space will be used (UDev, VDev), when the homography is scaled such as *h_33_* *= 1*. This standard deviation has been computed with the data obtained in many experiments with the real vehicles. From these results it can be stated that, in general, the computation of the relative position is very good if these deviations are lower than 1, good between 1 and 4, acceptable if they are between 4 and 7 and usually useless for values higher that 8. Notice that the correlation with the GPS has been intentionally discarded.

## 4. Planning for Multi-UAV FDI

As it has been mentioned in Section 1, since the accuracy and the noise level of the estimation depend on several factors, dynamic replanning of the multi-UAV mission is applied to improve the estimation. This replanning is based on the automatic generation of new tasks for the multi-UAV team, which are dynamically allocated during the execution of the mission. In this section, plans are only considered at a “high level”, i.e. each plan consists in a partially ordered set of high level tasks such as “visit a waypoint with a given heading”. Then, if a plan is composed by a list of “visit waypoint” tasks, the executive level of the UAV should compute straight paths between consecutive waypoints as the reference for its controller. Once a waypoint has been reached, the UAV stays in hovering until certain conditions are satisfied. Then, the UAV proceeds with the next waypoint in the list.

In general, the accuracy of the estimation is improved when the images of both UAVs are taken from points with similar altitude and camera orientation angles that differ less than 45°. Then, a suitable re-planning strategy is that the faulty UAV generates a task consisting in visiting a waypoint with a given camera orientation following this criteria and the safety constraints. Furthermore, a sufficient amount of texture in the images improves homography-based localization, and this can be used as an additional criterion to select the new waypoint.

This new task is generated considering safety issues, which are critical in multi-UAV missions. The best solution would be the implementation of sense-and-avoid techniques, but sometimes this is not possible due to payload limitations in small UAVs. In our approach, the conflicts between paths of different UAVs are solved at a waypoint level considering a safety radius around each UAV and a maximum separation with respect to the reference path. If a potential conflict is detected in a plan, it is solved with the suppression and addition of waypoints.

The initial trajectory planning takes into account a fixed inter-UAV safety distance, but when replanning is required, a different safety radius should be applied due to the uncertainties in the position of the faulty UAV. In our particular case, as the UAVs are helicopters, their hovering capabilities allow to simplify the conflict resolution problem when sharing the airspace. Once a fault has been detected, the UAV enters in a vision based hovering mode until another UAV provides additional information for the position estimation. In this mode, the UAV has a bigger (but bounded) safety radius for collision avoidance purposes. Then, the location of the waypoint satisfies the new safety radius constraints. Once an UAV has reached that place, it also stays hovering until the faulty UAV has landed.

During the experiments, the specific values for the safety radius in nominal and faulty conditions were 3 and 5 meters, respectively, and the location of the computed waypoints allowed to view the same region. At the executive level, under a maximum wind speed of 40 Km/h the UAVs were able to follow the paths between waypoints with errors below 0.5 meters.

The new task is dynamically inserted during the mission execution in the distributed task allocation framework applied in our multi-UAV architecture. It uses market-based negotiation rules implemented with a variant of the *Contract Net Protocol* (CNP) [17,18]. The main difference with the basic CNP protocol is that the bid of each UAV depends on its current plan and every time a local plan changes, the negotiation continues until no bids improve the current global allocation. When a new task is generated, all the UAVs take part in the negotiation of this task with the only restriction that the tasks in execution are not re-negotiated. The distributed algorithm allocates the new task trying to minimize its impact in the global cost of the mission (sum of the costs of all the UAVs).

If a fault is detected in the sensors of an UAV, the planning system will issue an emergency procedure to safely land the faulty UAV, helped with the position estimation of the other UAV. It is not safe to continue with the mission using the position estimation provided by the other UAV, because it will be correlated.

In order to illustrate the approach, let us consider a mission for three UAVs consisting in visiting five waypoints (see Figure 6a). After the initial distributed negotiation process, the allocation matrix is given by Table 1.

The execution starts and after visiting wp1, the UAV A sends a multi-UAV FDI request, and generates a new task consisting in visiting wp6 with a given camera orientation. UAV B and UAV C begin a negotiation process bidding with the insertion cost of the new task in their current local plans. As it can be seen in Figure 6b, this cost is lower for UAV B as far as wp4 is nearer from wp6 than wp3. Therefore, UAV B changes its local plan inserting task wp6 before task wp5.

Once that UAV B has reached the waypoint wp6, relative position estimation is performed using the algorithms presented in section 3 using the images from both cameras. This position estimation is fed to the fault detection system, along with the covariance estimation, with the corresponding threshold level.

## 5. Experimental Results

Experimental testing of fault detection systems is difficult because real experiments with faulty sensors or actuators can be very dangerous, particularly with aerial vehicles. One useful approach is to recreate offline experiments using real UAV flight data, introducing for example, a simulated fault in one sensor. Although it can not fully reproduce the experimental conditions, useful insights can be obtained from these experiments if faults are simulated properly. The results presented in this section have been done using real flight data and images obtained in experiments that were done in the framework of the COMETS project. In this project a distributed system for cooperative activities using UAVs was designed, implemented, and demonstrated for cooperative fire detection and localization. The objective is that a group of networked UAVs survey an area looking for fire alarms. If an alarm is detected, the team should localize it and confirm or discard it, taking benefit of the diversity of sensors. Since localization is one of the main objectives, UAV position accuracy is very important for the mission.

In this experiment, real flight data recorded by the helicopters *Marvin* and *Heliv* were used. *Marvin* is an autonomous helicopter developed by the Real-Time Systems & Robotics Group of the Technische Universität Berlin, Germany [19]. *Marvin* is built upon a conventional model airframe. Sensors for position and attitude determination include an IMU (with three magnetometers, three accelerometers, and three piezo-electric gyroscopes), an ultrasonic rangefinder looking down and a carrier phase differential GPS receiver. Marvin also has a digital camera.

*Heliv* is the result of the evolution of a conventional remotely piloted helicopter, adapted by the Robotics, Vision, and Control Group at the University of Seville (Spain) by adding sensing, perception, communication, and control functions. *Heliv* has an IMU, a GPS receiver and an ultrasonic altitude sensor for positioning, and it is equipped with one visual and one infrared cameras.

**Figure 7.**
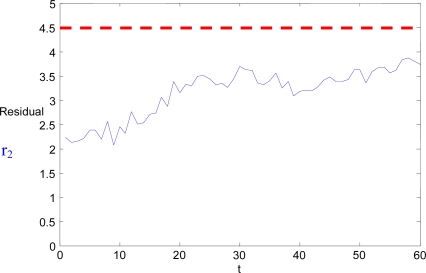
Residual r_2_ for position estimation sequence with initial standard deviations of UDev = 5.8 and VDev = 6.1, and threshold level of 4.5.

In the experiment carried out, *Marvin* and *Heliv* were instructed to patrol an area following their prescribed trajectories. A positioning error was artificially induced in the flight data recorded from *Heliv*. This error was a slow growing drift added to the position estimation. The fault detection of the individual UAV was not able to detect it. At fixed intervals, multi-UAV FDI tasks are executed, and Marvin is commanded to direct its camera to the same scene that Heliv is looking at. Following the procedure described in Section 3, the relative position of *Marvin* and *Heliv* is computed. The standard deviations of the homography are UDev = 5.8 and VDev = 6.1. The corresponding threshold level for this parameter is 4.5 (red dashed line in Figure 7). These variable threshold levels have been previously identified empirically, looking at the maximum value of the residual in fault-free conditions. An estimation of the absolute position of *Heliv* is generated using *Marvin* sensors. The discrepancies between this estimation and *Heliv*’s own estimation are shown in Figure 7, along with the threshold level corresponding to the standard deviations. No fault is detected, but, since the standard deviations of the homography are not very good, the mission planner generates dynamically a new task for *Marvin* to go to a new point nearer to *Heliv*, from which the homography of the taken images is expected to have better standard deviations.

**Figure 8.**
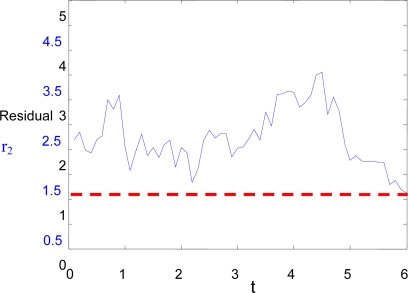
Residual *r*_2_ for position estimation sequence with initial standard deviations of UDev = 2.3 and VDev = 1.5, and threshold level of 1.5.

When *Marvin* arrives at this point, a new sequence of images is taken from both helicopters, and the relative position estimation is calculated again following the procedure from section 3. This time, the standard deviations of the homography are UDev = 2.3 and VDev = 1.5. Again, the discrepancies between this estimation and *Heliv*’s own estimation are shown in Figure 8, along with the threshold level corresponding to the standard deviations. In this case, the threshold level (dashed line) is lower, and it can be clearly detected that a fault is present in the sensors of the positioning system of *Heliv*.

## 6. Conclusions

Fault detection is an important issue in autonomous UAV navigation. Particularly, GPS transient failures, which are very usual in some scenarios, may have catastrophic effects. Computer vision can be used for relative position estimation in case of GPS failures. This paper has shown how computer-vision and task-replanning techniques can be used to improve the reliability in multi-UAV systems. The proposed method has been validated experimentally by using the information generated in the COMETS multi-UAV fire detection and monitoring field experiments. Future work will include the application of the proposed method by using cameras in fixed locations or transported by people and other sensors.

## Figures and Tables

**Table 1.** Initial Task Allocation Matrix.

	**wp1**	**wp2**	**wp3**	**wp4**	**wp5**
**UAV A**	1	1	0	0	0
**UAV B**	0	0	0	1	1
**UAV C**	0	0	1	0	0
